# Report on the Yellow Fever Epidemic at Wilmington, N. C., in the Autumn of 1862

**Published:** 1864-02

**Authors:** Wm. T. Wragg

**Affiliations:** Surgeon P. A. C. S.


### CONFEDERATE STATES MEDICAL & SURGICAL JOURNAL.

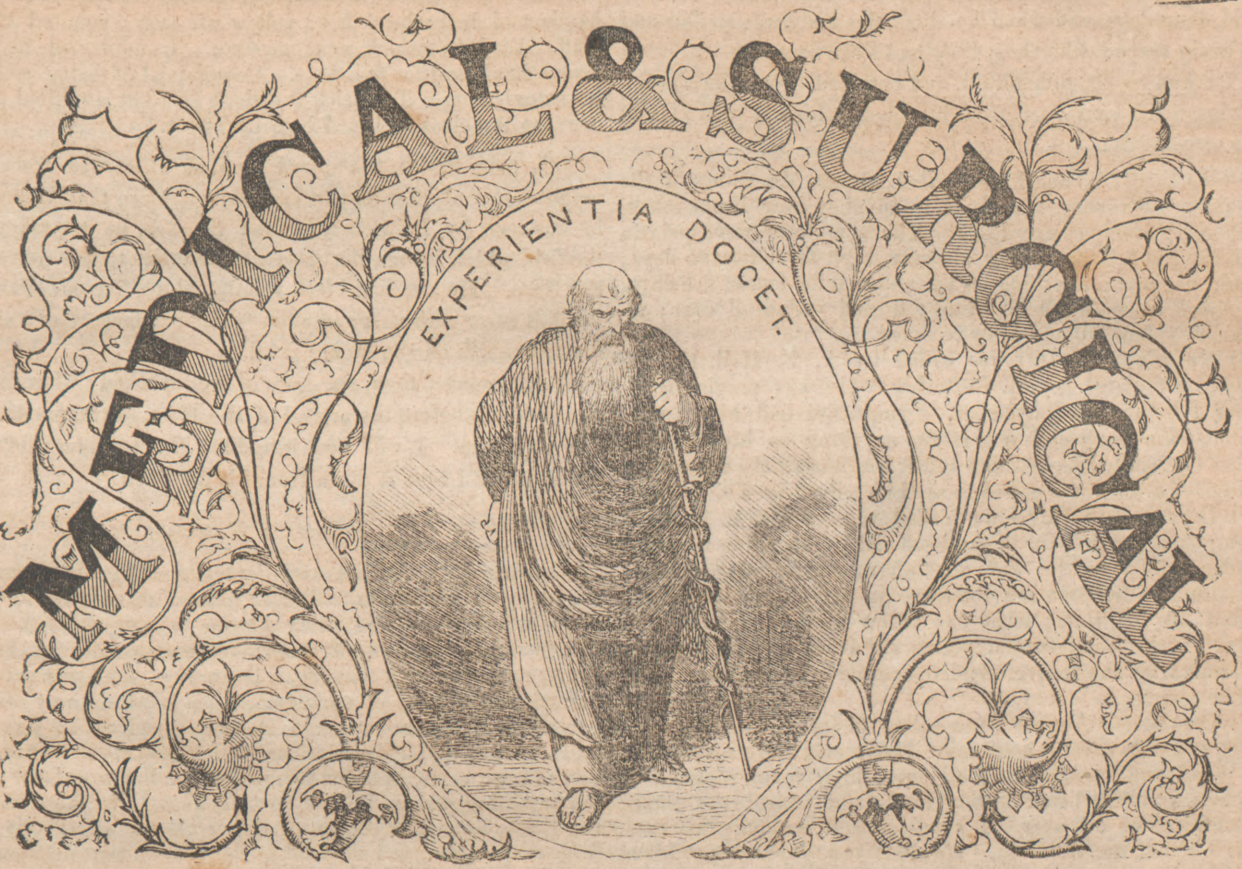

Vol. I. RICHMOND, FEBRUARY, 1864. No. 2.

### ORIGINAL COMMUNICATIONS.

Art. I.—*Report on the Yellow Fever Epidemic at Wilmington, N. C., in the Autumn of* 1862. By Wm. T. Wragg, Surgeon P. A. C. S.

[It is to be regretted that want of space compels us to abridge this interesting paper. Surgeon Wragg premises his report with a sketch of the topography of Wilmington. A sandy soil rests upon a sub-layer filled with water, so that slight depressions result in the formation of ponds which stagnate under the vertical rays of a tropical sun. These ponds were increased in size and number by the breastworks thrown up for the defence of the city, while the artificial drainage heretofore in use had, owing to the exigency of the times, been almost entirely neglected.

The hygienic condition of the city is described as being terrible in the extreme. The streets and yards were filled with heaps of filth and ordure, rotting in the sun. Disagreeable odors filled the air, requiring the constant use of tar-fires, whilst a dark canopy of smoke overhung the devoted spot. When we add to this the fact that many of the population were in want of the necessaries and comforts of life, living in the midst of this mass of decaying matter, we will not be surprised to hear that the physicians of the place stated that the season was unusually sickly, and that fevers of a malarial type, often terminating in jaundice, were becoming daily more prevalent.—Ed.]

*History of the Epidemic.*—Such was the state of things in and around the city of Wilmington when the Kate arrived at her wharf, near the foot of Market street, on the 6th of August, at 2 o'clock, P. M. Desiring to ascertain, as far as possible, what agency, if any, this arrival may have had on the origin of the yellow fever, I instituted a strict enquiry, both among the physicians and the inhabitants.

My first object was to know whether any cases of fever with symptoms like those of yellow fever (whether they had terminated fatally or favorably) had occurred before the arrival of the Kate. The first information was incidentally obtained from the inhabitants of one of the humble residences near the Rouse pond. This entire family (the Lamons) passed through the disease, one of them (Mrs. Nichols) dying with black vomit. I was informed here that they had been performing the last offices for those who had died around them for many weeks, and among others they mentioned Smith, the guard man, who had died about the 1st of August, under the care of Dr. Schonwald. He was sick, they said, four days, and when he died the body was yellow and in every respect like those they had since seen.

I called on Dr. Schonwald, and obtained from him the following notes, extracted from his book, which I will give in his own words:

"On the 22d June I was called to see Mr. Cling, of the German Volunteers, who lived in an alley near Front street, opposite the Cape Fear Bank. The symptoms were pain in the head and back, aching all over, high fever for two days; could not retain anything on the stomach for four or five days; inclined to vomit all the time; turned yellow all over; remained yellow till and after recovery.

"On the 26th July was called by A. E. Hall, Esq., to attend a mulatto boy, J. Lutterlow. He was previously attended by another physician. Symptoms: had high fever; yellow all over; could not retain anything on his stomach; what he vomited looked like snuff; restless all the time; died two days after I was called to see him; lived in Third street.

"On the 30th July I was called to see Mrs. E. Hausley. Symptoms, severe pain in the head and back, aching in all the limbs, high fever for two days after; she could not retain anything on her stomach for three or four days; inclined to vomit all the time; what she threw up looked like snuff mixed with blood; turned yellow all over; remained yellow till and after she recovered; lives corner of Seventh and Red Cross streets.

"On the 4th August I was called to see Lieut. Davidson, of Newkirk's cavalry company. Symptoms were severe pain in head and back, aching all over, high fever, inclined to vomit; vomited black bloody stuff, and passed the same; turned yellow all over. I considered it a dangerous case. Dr. Thomas was called in. He died two days after. Lived in Third street, north of Market.

"On the 5th August I was called to see a girl boarding at Mrs. McLin's. Her name was Georgia Weeks. It was a house of ill-fame. Symptoms, severe pain in head and back; vomiting black stuff; could not take medicine or any nourishment; vomiting all the time; yellow all over. Died next morning. Was sick two days before I saw her. Lived in Water street in a house called Hole-in-the-Wall."

Four out of five of these cases, it will be seen, threw up black vomit, and all of them antedated the arrival of the Kate, and though they occurred in the practice of one not recognized by the medical fraternity of Wilmington as one of the profession, they are, nevertheless, entitled to be considered as facts for record and examination. It was well known in Wilmington that they had occurred. The symptoms they presented were the subject of conversation, and one of them (that of Lieut. Davidson) finally terminated under the care of Dr. Thomas, one of the most distinguished physicians of the place.

It is very much to be regretted that the illness and absence from Wilmington of this gentleman prevented me from obtaining in writing the valuable information he possessed in relation to this subject. I may state here, however, that, in conversation with me, he gave me many details entirely corroborative of the statements of Dr. Anderson in relation to the sanitary condition of Wilmington, which statements will be given in the very words of that accomplished physician.

Dr. Schonwald goes on as follows:

"On the 8th of August I was called to visit a man named Robert Smith, town guard, and a butcher. Symptoms cold hands and feet; head hot; yellow all over; vomited black stuff. Died 9 o'clock next morning. I understood he had been stimulating for five days. Complained of being sick in his intoxication. Took sick 9 o'clock at night, and died next morning 9 o'clock. Lived in Fifth street."

This man Smith, the guard man, as I learned from his neighbors, died after an illness of four days, so that, although he was seen by Dr. Schonwald on the 8th, he was taken on the 5th, which brings his case also anterior to the arrival of the Kate. He also had black vomit, making five out of six of Dr. S.'s cases with that symptom. His residence was a short distance north of the Rouse pond.

We have here, then, six cases of well marked yellow fever, all occurring before the arrival of the Kate, in the practice of one physician. I will now introduce the testimony of Dr. Anderson as I took it down from his dictation.

*Dr. Anderson's Statement.*

"During July, August and September the entire country was deluged with rain. Ponds formed on high and dry places where water was never known to accumulate before, and, owing to neglect of the culverts, especially on Front street, near Robert's foundry, a large long, shallow pond was formed, the bottom of which was composed entirely of the sweepings of the streets—old shoes, rags, pieces of tin, and refuse matter of all descriptions, which had been thrown in by the town carts, in order to raise the valley to a level with the adjoining streets. This spot is known by the name of the Rouse lot. The bottom of this pond was alternately dry from evaporation, and exposed to the intense heat of the sun, and then again filled by fresh rains, when it was covered by green slime, and exhaled a most offensive odor. One square south of this pond, in the direction of the prevailing winds, the first cases of fever commenced. On the first day of August a young man in the employment of Government, by the name of Morris, was taken sick, with all the symptoms of yellow fever, and recovered after a protracted illness.

"Three squares east of the Rouse pond, on Fourth street, another pond formed in the immediate vicinity of a house occupied by Mrs. Johnson, who died with black vomit, and had two children ill with yellow fever. These cases occurred about the 12th of August. One square south of the same pond the case of Smith, the guard man, occurred on the 5th August. Around these two ponds many fatal cases of the disease have continued to occur during the whole course of the epidemic.

"In the Campbell house, one door north of the Rouse pond, several fatal cases occurred; and this quarter of the town has been more afflicted than any other.

"On Market street, commencing at the Carolina Hotel, on the north side, there is a range of cellars which have been covered at least one foot deep with water, and though frequently bailed out, continued to be flooded, and are still filled with water. Most of these cellars are unused, but are more or less filled with accumulated trash, scraps of old leather and decayed harness. Four of the druggists' stores are over these cellars, and floating about in the water were straw from old hampers of wine and medicines, leaky casks of oil, varnish, acids and paint. One could hardly walk in these cellars without having the water coming over the tops of the boots; and in the open area in front, where the water was exposed to the sun and light, a thick coating of green slime floated on the surface.

"One of the druggists occupying these stores was among the earliest victims of the disease; and from another two more were buried in succession; and from the third both the principal and assistant were taken down with the disease; and the occupant of the fourth was also ill. Indeed, for several days there was no druggist in Wilmington, and the stores had to be supplied from Mobile, Charleston and Savannah.

"On the south side of Market street, just opposite to these drug stores, many fatal cases occurred, and scarcely one of the inhabitants of this section of the city escaped an attack, which, in most cases, proved fatal.

"On that part of Water street lying north of Market there are no cellars to the buildings, but the floors are, for the most part, raised two feet from the ground, with free ventilation by means of perforated copper-plates let into the wall. The incessant rains had flooded the earth beneath these floors to such an extent that the water rose above the joists and splashed up through the cracks of the floor as persons walked over them. These stores were occupied by small retail dealers, mostly Germans, and it was notorious that the earliest cases of the fever were among them.

"The deluging rains were succeeded by an unusually high range of the thermometer with most scorching heat of sun, almost insupportable by man, and even by the animals of labor. The city was enveloped in vapor from this heat and dampness, and at the same time the dew point was unusually high. The leaves were covered with moisture as after a heavy rain. Everything was covered with mould—even the books in the libraries and clothes in the presses. Shoes cleaned over night were mouldy in the morning. The goods in the stores were in the same condition.

"Owing to the absence of the population in consequence of the fear of an attack on the city, and the attention of the authorities being turned to the defences of the place, little or no scavengering had been done for some time, so that the more populous portions of the city were in a very dirty condition, with large quantities of decaying offal collected in the yards. This was particularly the case in the Marcus Hotel, at the corner of Market and Front streets, and also the Palmetto, a few doors north of market street, in both of which early and malignant cases of yellow fever occurred.

"On the eastern border of the town, at the distance of about half a mile from the limits, there is an extensive mill-pond, long and narrow, and facing the whole of that side of the town. It covers from 50 to 100 acres of land. About the latter part of August, in the midst of the heavy rains, the dam broke and emptied the contents of this extensive pond to the depth of five or six feet, laying bare large quantities of rotting wood and extensive margins of slimy soil. From this denuded surface there arose a most offensive odor, which was wafted directly towards the city by an unusual prevalence of easterly wind. The skirt of woods which intervened between this pond and the city had just been cut down to allow the guns, placed in position on its city side, to play, so that there was no impediment to the progress of the malaria. The earth has been broken up all around the city for the construction of fortifications, thereby exposing extensive surfaces of fresh soil Nearer to the city, and, indeed, within its limits, around the Marine Hospital, which is built on a high, hilly, and usually, perfectly dry spot, the heavy rains have caused the water in the little valleys to rise and fall, alternately, above and below the surface of the earth as the rain was more or less abundant. And near by this same region, on the lot occupied by the State stables, is an extensive pond, covered by green vegetation, into which the refuse of the stables has been deposited for many months, producing a most offensive state of things.

"This region has been occupied for many months by encampments of soldiers, by whom much filth and refuse have been collected in the by-places.

"The health of this part of the town, which in ordinary seasons has been very good, has this year been very severely handled by the epidemic. These causes of disease, it would appear, have been producing an unhealthy state of the atmosphere for many weeks previous to the appearance of yellow fever. Camp measles, dysentery and typhoid, in an aggravated form, were very common. Jaundice had been epidemic. It has been estimated that about one-fourth of the remaining population, and the soldiers had the disease, which in many cases proved fatal. Many cases, at the time supposed to have been jaundice, are now believed to have been early cases of yellow fever.

"Under this state of things in Wilmington, the steamer Kate arrived from Nassau on the 6th of August, which port she left about the 2d. Captain Lockwoood stated to gentlemen in Wilmington that the yellow fever had been carried into Nassau by the ship Flying Cloud, from Havana, which port she left early in July, loaded with coal, having reached Nassau about the middle of the same month. Soon after the arrival of the Flying Cloud in Nassau, the British steamer Kersonese took her place to leeward of her. The Flying Cloud had the disease on board at that time, and it was soon communicated to the crew of the Kersonese, several of whom died of it on the passage of that ship to Bermuda. It was in the same way communicated to other vessels in the port of Nassau, and finally to the inhabitants of the town; this being the first time it has ever been known to have been epidemic in that place.

"The Kate, in her pasage out, was chased by a Federal steamer for several days. The fires in the furnaces were driven to the utmost extent that prudence allowed. Very little wind prevailed, and what did blow was aft, so that the circulation of air on board of the vessel was very imperfect. The engineer states that at the time of the chase, and up to the arrival in Wilmington, the vessel was so much heated from the fires in the furnaces, that the thermometer stood at 140° in the saloons, gangways and state-rooms; that the woodwork was so much heated that it was impossible to bear the hand pressed upon it; that in the fire and engine rooms the thermometer stood at 180°; the men worked in nothing but their drawers, and were bathed in perspiration; and this heated condition of the vessel was kept up for several days after her arrival in the port of Wilmington consequent upon the intense heat of the atmosphere there.

"The vessel arrived in Wilmington at 2 o'clock P. M., August the 6th, one of the hottest and most oppressive days of the season. Immediately after arrival, Dr. Anderson was called to see O'Donohoe, one of the firemen, who had been taken sick the day before, being the third day out from Nassau. His symptoms were intense headache, injected eyes, pain in the back, nausea and vomiting. In his delirium that night he strayed away from the ship and Dr. Anderson saw him no more. After rambling about for thirty hours, he was taken into the Marine Hospital on the morning of the 8th, where he died in a few hours.

"On the evening of the 16th August, Dr. Anderson was called to visit a seaman, named Dennis Mitchel, of the Kate, in the house of a man named Campbell, 400 feet north of the Rouse pond above described. He had been sick for several days on board the Kate while lying at the Marine Railway near by. This patient had all the symptoms of a violent attack of yellow fever. He was delirious, and refused medicine. On the morning of the 17th (the next day) the body was laid out ready for interment, while the adjoining room was fillep with spectators of both sexes examining the body and indulging in a carousal Three or four days after the death of Mitchel, Campbell, the proprietor of the house, his wife and children, all died of the disease, as did, also, many of the other spectators.

"At the same time, on the opposite corner of the square, Mrs. Peterson was attacked and recovered, but two inmates of her house have since died.

"On the 21st of August, Dr. A. was called to see Mr. Egan, chief mate of the Kate, on board the vessel which had by that time left the Marine Railway, after having cleansed and prepared for the reception of her outward cargo. On the same evening he was removed to the Palmetto Hotel, where he was kindly attended by the proprietor, Mr. Bailey and his son and other members of the family. On the 24th, the Kate sailed with Mr. Egan on board, convalescent. Mrs. Bailey and her son have both died of the disease, having been attacked, the latter in the beginning of October, and the former about the middle.

"Before leaving the history of the Kate it may be stated that she was discharged by soldiers detailed from regiments stationed near the city, and, so far as can be ascertained, no case of yellow fever has occurred among them, nor among any of the partners or employees of the firm to whom she was consigned, as was positively affirmed by one of the partners.

"On the 15th of August, Mr. Thomas Holden was attacked with all the smyptoms of yellow fever. He lived at least half a mile from where the Kate lay, having had no communication whatever with her.

"On the 28th August, Dr. A. saw Walter Furlong, who had all the symptoms of yellow fever. He lived near the Catholic church, in a central part of the town, having had no communication with the Kate, or any of the places where her sick had been. He recovered.

"From this date the disease increased rapidly in the practice of Dr. A. and the other physicians of Wilmington, and on the 14th September, the official announcement was made that it was epidemic. On the 28th, Dr. Dickson, one of the most distinguished physicians of the State, fell a victim to the scourge, and soon after, Drs. Thomas, Swann and Love were attacked, and after severe illness, happily recovered. At this time there were only three out of eight resident physicians left for duty."

Here Dr. Anderson's highly important statement ends. It will be observed that the case of the young man Morris, in Government employment, is mentioned as having occurred on the first of August, one square south of the Rouse pond, making the seventh for which the Kate cannot be held responsible; while the cases of Mrs. Johnson and her two children, in the vicinity of the same pond, being only three squares east of it, and adjoining another pond, occurred *about* the 12th. If they *commenced* on the 12th, they were only six days after the arrival of the vessel and while she was half a mile off in a direction against the prevailing winds; and if they *terminated* at the time stated, they must have sickened before the vessel arrived.

On the 6th August, the day the Kate arrived, Dr. Anderson saw O'Donohoe on board. He left the vessel that night in his delirium, and after wandering about for thirty hours, was taken into the Marine Hospital on the morning of the 8th, where he died the same day. There was, no doubt, in the minds of any who saw him, of the nature of his disease. Let us see if he communicated it to the inmates of the institution.

By the kindness of Surgeon Custis, of that hospital, I obtained from the books of the house the following facts: The next death that occurred after O'Donohoe's was that of Dixon, who entered the hospital on the 20th of August, twelve days after the death of O'Donohoe, having been ill for some days previous. "He had daily paroxysms of chill and fever, and died on the 26th of August, in a congestive chill." The next death was that of Veach, on 28th August, "of remittent fever." The next was that of Getty, and "his was the first case of black vomit. He was admitted on the 28th September. He recovered. He had been in Wilmington doing provost guard duty."

This man, Getty, it will be remarked, entered the hospital fifty-one days after the death of O'Donohoe. It will, also, be noticed, that he had been doing exposed duty in Wilmington after the yellow fever had been officially declared epidemic.

After this time, other cases of yellow fever were admitted and treated in the hospital, and Surgeon Custis informed me that they all had been constantly about the streets of Wilmington, on duty or otherwise.

The next point to which my inquiries were turned, was to ascertain if the disease had spread beyond the immediate limits of Wilmington—in other words, whether any well authenticated cases could be cited in individuals who had not been in Wilmington. Cases had occurred among the refugees at several of the settlements on the Sounds, at various places in the country residences around, at points on the Cape Fear river, above the city of Wilmington, among the camps near and at a distance, and finally, at Smithville, near the mouth of the river. After the most careful and diligent investigation, I could not learn that even a suspicion of its having been propagated among those who had not breathed the tainted air of the infected city, was entertained in regard to any of these places except Smithville. Of the earlier deaths at that place, every one was known to have been in Wilmington, and I learn from Prof. T. G. Prioleau, who spent some weeks there during the greatest prevalence of the disease, that though many of the inhabitants attributed the sickness of their town to communication with Wilmington, (as Wilmington to Nassau) yet, so far as he could learn, every individual attacked in Smithville, had been in Wilmington at some time during the sickly season; and though some few may not have been there for six or eight weeks anterior to their illness, yet most of them had been there much later. It is reasonable to suppose that this statement is in strict accordance with the facts, when we reflect that there was daily intercourse between the places by land and water.

With this historical statement of the circumstances connected with the appearance, extension and limitation of the disease, the reader will be in possession of all the data requisite for forming his own opinion as to its origin.

[TO BE CONCLUDED IN NEXT NUMBER.]

